# Supplementary material for: Exploring the Relationships between Lifestyle Patterns and Epigenetic Biological Age Measures in Men
Source: Biomedicines. 2024 Sep 2;12(9):1985. doi: 10.3390/biomedicines12091985 (PMC11428654; doi:10.3390/biomedicines12091985)
Supplement: Supplementary file 1 [file biomedicines-12-01985-s001.zip › biomedicines-3124736-supplementary.pdf]

Table S1. Classification of the lifestyle-related exposures.

| | Variable | Groups | Note |
|---|---|---|---|
| 1. | Body mass index (BMI) | Normal (18.5 ≤ BMI < 25) | The information was collected from the screening questionnaire. The classification was divided into three groups based on NHS suggestion [82]. |
| | | Overweight (25 ≤ BMI < 30) | |
| | | Obese (BMI ≥ 30) | |
| 2. | Tobacco smoking | Never | The information was obtained from Lifestyle Pattern Questionnaire. |
| | | Previous | |
| | | Current | |
| 3. | Alcohol intake amount | Never | The information was obtained from Lifestyle Pattern Questionnaire. Alcohol consumption used the threshold of 7 drinks a week for categorisation. A drink was assumed to be one can or bottle of beer, or a glass of wine. |
| | | < 7 drinks/week | |
| | | ≥ 7 drinks/week | |
| 4. | Physical activity | No exercise habit | The information was obtained from Lifestyle Pattern Questionnaire. |
| | | Exercising occasionally | |
| | | Exercising routinely (at least 3 times a week, 30 minutes a day) | |
| 5. | Cholesterol intake | <300 mg/d | The information was obtained from Food Frequency Questionnaire. Although the 2015–2020 Dietary Guidelines for Americans (DGA) removed the recommendation to restrict dietary cholesterol to 300 mg [83], the 2020-2025 DGA still advises that dietary cholesterol consumption should be as low as possible [84]. Additionally, foods that contribute most to cholesterol intake are usually high in saturated fats. Furthermore, well-known healthy eating patterns such as the Mediterranean-style [85] and DASH-style diets [51] also advocate for low cholesterol consumption. Therefore, this study continued to adhere to the earlier suggestion by the National Institute for Health and Care Excellence (NICE) that individuals at risk of cardiovascular disease in the UK should not exceed 300 mg of cholesterol per day [47,38]. |
| | | ≥300 mg/d | |
| 6. | Saturated fat intake | Men <30 g/d, Women <20 g/d | The information was obtained from Food Frequency Questionnaire. The saturated fat threshold was set at 30g per day, following the NHS recommendation of no more than 30g for men and 20g for women per day [39]. |
| | | Men ≥30 g/d, Women ≥20 g/d | |
| 7. | Total sugar intake | ≥90 g/d | The information was obtained from Food Frequency Questionnaire. Total daily sugar |

| | Variable | Groups | Note |
|---|---|---|---|
| | | <90 g/d | intake per day was classified with a threshold of 90g daily, adhering to the NHS recommendation not to exceed this value [40]. |
| 8. | Vitamin D intake | ≥10 mcg/d | The information was obtained from Food Frequency Questionnaire. Vitamin D categories were based on the NHS recommendation of at least 10 mcg/day [41]. |
| | | <10 mcg/d | |
| 9. | Daily calorie intake | Men <2500 kcal/d, Women <2000 kcal/d | The information was obtained from Food Frequency Questionnaire. The daily calorie intake threshold was set at 2500 kcal/day for men and 2000 kcal/day for women, as the NHS suggests not exceeding these values [42]. |
| | | Men ≥2500 kcal/d, Women ≥2000 kcal/d | |

Table S2. The component of healthy lifestyle index (HLI).

| **Variables** | **Category** | **Score** | **Variables** | **Category** | **Score** |
|---|---|---|---|---|---|
| **BMI** | Normal | 3 | **Cholesterol intake** | <300 mg/d | 2 |
| | Overweight | 2 | | ≥300 mg/d | 1 |
| | Obesity | 1 | **Saturated fat intake** | Men <30 g/d, Women <20 g/d | 2 |
| **Smoking** | Never | 3 | | Men ≥30 g/d, Women ≥20 g/d | 1 |
| | Previous | 2 | **Total sugar intake** | <90 g/d | 2 |
| | Current | 1 | | ≥90 g/d | 1 |
| **Alcohol** | Never | 3 | **Vitamin D intake** | ≥10 mcg/d | 2 |
| | < 7 drinks/week | 2 | | <10 mcg/d | 1 |
| | ≥ 7 drinks/week | 1 | **Daily calorie intake** | Men <2500 kcal/d, Women <2000 kcal/d | 2 |
| **Physical activity** | Keep exercising routinely | 3 | | Men ≥2500 kcal/d, Women ≥2000 kcal/d | 1 |
| | Exercise, but not routinely | 2 | | | |
| | No any exercise | 1 | | | |
| **Total score** | | 10-22 | | | |

**References**

82 Body Mass Index from NHS. Available online: https://www.nhs.uk/common-health-questions/lifestyle/what-is-the-body-mass-index-bmi/. (accessed on 13 February 2024).

83 Soliman, G.A. Dietary Cholesterol and the Lack of Evidence in Cardiovascular Disease. Nutrients **2018**, *10*, 780, https://doi.org/10.3390/nu10060780.

84 Dietary Guidelines for Americans 2020-2025. Available online: https://www.dietaryguidelines.gov/sites/default/files/2021-03/Dietary_Guidelines_for_Americans-2020-2025.pdf (accessed on 30 March 2024).

85 Rees, K.; Takeda, A.; Martin, N.; Ellis, L.; Wijesekara, D.; Vepa, A.; Das, A.; Hartley, L.; Stranges, S. Mediterranean-style diet for the primary and secondary prevention of cardiovascular disease. Cochrane Database Syst. Rev. **2019**, *2019*, CD009825, https://doi.org/10.1002/14651858.cd009825.pub3.

51 Appel, L.J.; Moore, T.J.; Obarzanek, E.; Vollmer, W.M.; Svetkey, L.P.; Sacks, F.M.; Bray, G.A.; Vogt, T.M.; Cutler, J.A.; Windhauser, M.M.; et al. A clinical trial of the effects of dietary patterns on blood pressure. DASH Collaborative Research Group. N. Engl. J. Med. **1997**, *336*, 1117–1124, doi:10.1056/nejm199704173361601.

47 NHS:Cholesterol Available online: https://www.oxfordhealth.nhs.uk/wp-content/uploads/2014/08/OP-026.14-Cholesteral.pdf (accessed on 29 March 2024).

38 Evidence review for dietary cholesterol strategies. Available online: https://www.nice.org.uk/guidance/ng238/evidence/b-dietary-cholesterol-strategies-pdf-13253901662 (accessed on 27 March 2024).

39 Fat: the facts. Available online: https://www.nhs.uk/live-well/eat-well/food-types/different-fats-nutrition/#:~:text=Saturated%20fat%20guidelines,of%20saturated%20fat%20a%20day (accessed on 27 March 2024).

40 Sugar: the facts. Available online: https://www.nhs.uk/live-well/eat-well/food-types/how-does-sugar-in-our-diet-affect-our-health/#:~:text=The%20reference%20intake%20for%20total,more%20information%2C%20see%20Food%20labels. (accessed on 27 March 2024).

41 Vitamin D. Available online: https://www.nhs.uk/conditions/vitamins-and-minerals/vitamin-d/ (accessed on 27 March 2024).

42 Calorie counting. Available online: https://www.nhs.uk/better-health/lose-weight/calorie-counting/#:~:text=How%20many%20calories%20to%20eat,2%2C000kcal%20for%20women (accessed on 27 March 2024).
